# The Correlation between Traditional Chinese Medicine Constitution and Hyperuricemia and Gout: A Systematic Review and Meta-Analysis

**DOI:** 10.1155/2023/5097490

**Published:** 2023-04-17

**Authors:** Zihao Jiang, Jieyu Chen, Yanting You, Shuai Ji, Liqian Chen, Qiuxing He, Yanyan Liu, Xiaomin Sun, Lin Zhou, Xiaoshan Zhao

**Affiliations:** ^1^Syndrome Laboratory of Integrated Chinese and Western Medicine, School of Chinese Medicine, Southern Medical University, Guangzhou, China; ^2^Endocrinology Department, Nanfang Hospital, Southern Medical University, Guangzhou, China

## Abstract

**Objective:**

To investigate the correlation between the constitution of traditional Chinese medicine (TCM) and hyperuricemia (HUA) and gout.

**Method:**

Databases including China National Knowledge Infrastructure (CNKI), WanFang Data, China Science and Technology Journal Database (VIP), China Biology Medicine Disc (CBMdisc), PubMed, The Cochrane Library, Web of Science, and Excerpta Medical Database (Embase) were searched to collect observational studies about TCM constitution in HUA and gout from inception to November 21, 2021. The distribution of TCM constitution types in HUA and gout patients was presented by proportion, while the correlation was presented by odds ratio (OR) and 95% CI. Meta-analysis was performed using StataCorp Stata (STATA) version 16.0 software.

**Results:**

Twenty-one cross-sectional studies and 10 case-control studies involving 38028 samples were included, among which 27526 patients were diagnosed with HUA and 2048 patients with gout. Phlegm-dampness constitution (PDC), damp-heat constitution (DHC), and qi-deficiency constitution (QDC) are the most common types, accounting for 24% (20%–27%), 22% (16%–27%), and 15% (12%–18%), respectively, in HUA patients, while DHC, PDC, and blood stasis constitution (BSC) accounted for 28% (18%–39%), 23% (17%–29%), and 11% (8%–15%), respectively, in gout patients. PDC and DHC were the main constitution types in patients with HUA or gout in south China, east China, north China, southwest China, northwest China, and northeast China. There was no difference in the distribution of PDC and QDC in male or female patients with HUA, while males with DHC in HUA were more common than females. The proportion of PDC or DHC among HUA patients was 1.93 times and 2.14 times higher than that in the general population (OR and 95% CI: 1.93 (1.27, 2.93), 2.14 (1.47, 3.13)), while the proportions of PDC, DHC, and BSC were 3.59 times, 4.85 times, and 4.35 times higher than that of the general groups (OR and 95% CI: 3.59 (1.65, 7.80), 4.85 (1.62, 14.57), and 4.35(2.33, 8.11)).

**Conclusion:**

PDC, DHC, and QDC are the main constitution types of patients with HUA, while PDC and QDC may be the risk factors for HUA. DHC, PDC, and BSC are the main constitution types of patients with gout, and they may be the risk factors for gout. In clinical and scientific research, more attention should be paid to the relationship between the above-mentioned TCM constitution in HUA or gout. Nevertheless, because the quality of the included observational studies is low, more prospective cohort studies related to TCM constitution and HUA or gout can be carried out to verify the causality between TCM constitution and HUA or gout.

## 1. Introduction

Hyperuricemia (HUA) refers to an abnormally high concentration of serum uric acid (SUA), typically defined as >420 *μ*mol/L twice on a different day [1]. When the level of SUA is too high, uric acid crystals are deposited in the joints, causing inflammation and tissue destruction, which is called gout. The prevalence of HUA continues to increase and shows a trend in the world. As of 2019, the prevalence of HUA was up to 17.4% and 16.6% of the population in China [2] and Australia [3], respectively. Men are more likely to get HUA than women [3]. HUA is the main reason for gout, and elevated uric acid in the serum is associated with serious metabolic diseases and cardiovascular diseases such as chronic kidney disease, hyperlipidemia, type 2 diabetes, hypertension, heart diseases, and many other diseases [4, 5]. HUA has become another common chronic metabolic disease after diabetes. The prevention and cure of HUA and gout have also become major problems that remain to be solved at present.

Traditional Chinese medicine emphasizes preventative treatment, that is, identifying the disease before its onset or preventing it from developing into a pathological state. The TCM constitution is an important entry point for preventative treatment in TCM [6]. The TCM constitution is a stable and integrated intrinsic characteristic of our body in physiological functions, morphological structure, and psychological state, which is the outcome of both genetic and environmental factors. According to modular theory, the TCM constitution can be divided into 9 basic types: yang-deficiency constitution (YADC), qi-deficiency constitution (QDC), qi-stagnation constitution (QSC), phlegm-dampness constitution (PDC), yin-deficiency constitution (YIDC), damp-heat constitution (DHC), blood stasis constitution (BSC), balance constitution (BC), and inherited special constitution (ISC). Among them, BC has a normal constitution, while the others have different characteristics in disease tendency, physiological and pathological states, and other aspects. Based on the TCM constitution, the systems of three-level prevention, the system of Screening-Warning-intervention For Chronic Disease, and the system of Early Prediction-Early prevention-Early intervention have gradually become a precision medicine model with TCM theory.

With the recognition of the TCM constitution identification into the national public health service system and the China Mid- and Long-term Plan for Prevention and Treatment of Chronic Diseases (2017–2025), studies on the correlation of the TCM constitution with HUA or gout have been carried out nationwide, but there has been no systematic review yet. Thus, a systematic review is needed to integrate the enormous clinical research, summarize the distribution of TCM constitution for HUA or gout treatments, and find out the exact correlation between TCM constitution and HUA or gout. The results will come up with a perspective for risk population identification, classified treatment, and prognosis prediction of HUA and gout in the clinical and scientific fields.

## 2. Materials and Methods

### 2.1. Registration

The protocol was registered at PROSPERO (CRD42021292561) on November 21, 2021, accessible at https://www.crd.york.ac.uk/prospero/display_record.php?RecordID=292561.

### 2.2. Searching Strategy

We searched CNKI, WanFang Data, VIP, and CBMdisc 4 Chinese databases with “hyperuricemia,” “gout,” and “constitution” as the subject terms and “Chinese medicine” as the full text or unlimited fields. We searched Pubmed, Web of Science, Cochrane Library, and Embase 4 databases with “hyperuricemia,” “gout,” “gouts,” “constitution,” and “Chinese medicine” as all fields. We reviewed literature published from the day the database was founded to November 21, 2021, and did not set language restrictions on literature. The search strategies used in the above English databases are presented in Supplementary Table S1.

### 2.3. Eligibility Criteria

#### 2.3.1. Types of Trials

Cross-sectional studies, case-control studies, and cohort studies.

#### 2.3.2. Types of Participants

Patients diagnosed with HUA, patients diagnosed with gout, and reports should have clear diagnostic criteria for HUA or gout, and there is no limitation on age, gender, and course of the disease.

#### 2.3.3. TCM Constitution Recognition Tool

The TCM Constitution is based on *The Classification and Determination of Traditional Chinese Medicine Constitution* issued by the Chinese Society of Traditional Chinese Medicine [7].

#### 2.3.4. Outcomes

Studies that recorded the distribution of basic types of TCM constitution in patients with HUA or gout or studies that recorded the comparative data between the proportions of TCM constitution in HUA or gout patients and the proportions of TCM constitution in the general population were included for further analysis.

#### 2.3.5. Exclusion

The exclusion criteria were as follows: (1) studies that did not report the basic information of participants, such as age and gender. (2) Patients included in the studies had serious diseases that may affect the TCM constitution, such as diseases in the cardiovascular, cerebrovascular, liver, kidney, endocrine, or hematopoietic systems. (3) Reports that used duplicate samples from the same population or reports that had deficient data were excluded.

### 2.4. Data Extraction and Quality Assessment

Two researchers independently screened the literature and extracted the following information: (1) The basic information of the studies, including the research topic, the author's name, the presentation time, etc. (2) Baseline information about the objects, including gender and average age. (3) Areas where studies were carried out. (4) The time that studies started and ended. (5) Number of cases and types of the reported TCM constitution. (6) Key elements of the quality assessment. The quality of the included cross-sectional studies was assessed under 11 items, with the highest mark of 11 published by The Agency For Health Care Research and Quality (AHRQ) [8]. As for the results of the AHRQ, 0–3 is considered low quality, 4–7 is considered medium quality, and 8–11 is considered high quality. Case-control studies were assessed by the Newcastle-Ottawa Scale (NOS), including 8 items with the highest mark of 9 [8]. Any mark in NOS over 6 is considered high quality.

An individual may be reported with 2 or more than 2 types of TCM constitution at the same time, so if an individual were reported to be with 2 or more than 2 types of TCM constitution at the same time, the individual would be counted in the number of every single type of TCM constitution. For example, if 15 individuals were reported DHC, and 10 individuals were reported QDC, and there were 10 individuals who reported both DHC and QDC, then 25 numbers of DHC and 20 numbers of QDC were included.

### 2.5. Statistical Analysis

Statistical analysis was performed with the software STATA version 16.0. The meta-analysis was conducted in two parts. The first part was to conduct a meta-analysis based on cross-sectional studies or case-control studies that reported numbers or events on proportions of different types of TCM constitution in patients with HUA or gout patients. The effect values were calculated with a single rate and 95% confidence. The results of the three TCM constitutions with the highest proportion among the nine types of TCM constitutions were presented in forest maps, and the results of the rest of the TCM constitutions were listed in a table.

The second part was to conduct a meta-analysis based on case-control studies that reported comparative data between the distribution of TCM constitutions in HUA or gout patients and the distribution of TCM constitutions in the general population. The effect values were calculated as odds ratio (OR) and 95% confidence interval (95% CI). Comparative results of the distribution of TCM constitutions between HUA or gout patients and general groups were all listed in another table.

Heterogeneity was assessed by *I*^2^ statistics. If *I*^2^ < 50% and *P* > 0.10, indicating relatively low heterogeneity, then a fixed effects model was chosen. If *I*^2^ > 50% and *P* < 0.10, indicating relatively high heterogeneity, then a random-effects model was applied and subgroup analysis was applied according to regions and genders. Sensitivity analysis was used to assess the stability and reliability of the results of the meta-analysis. For the results of more than 10 studies included, funnel plots and Egger's test were applied to evaluate the potential publication bias. The level of the meta-analysis was set to *α* = 0.05.

## 3. Results

### 3.1. Literature Search

**Figure 1:**
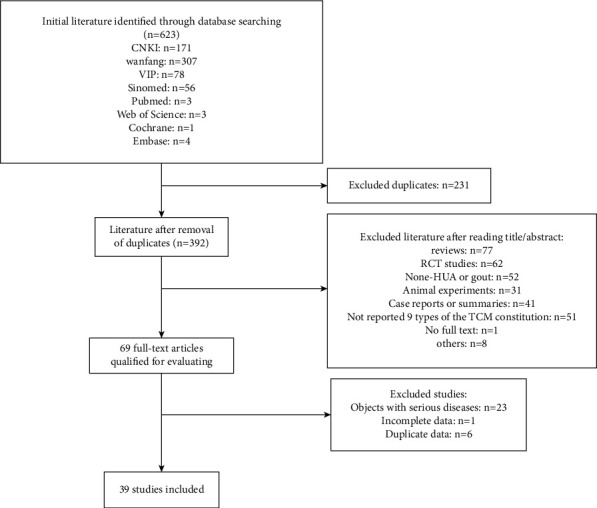
The flow chart of study selection.

Following the search strategy, the initial search yielded 623 studies, including 612 in Chinese and 11 in English. After duplicates were removed, 392 articles were retrieved for further assessment. And by title and abstract screening, followed by the above selection criteria, 39 studies were finally included [9–46] (Figure 1), with 19 cross-sectional studies [10–17, 19–21, 23, 24, 26,27,29,30,32,33] on HUA, 9 cross-sectional studies about gout [18, 34–37, 39, 41, 43, 44], 6 case-control studies about HUA [9, 22, 25, 28, 31, 45], and 4 case-control studies about gout [38, 40, 42, 46], and 1 cross-control study [18] on both HUA and gout. The fundamental information of the studies is shown in Table 1. The number of people and comparative data of each type of TCM constitution can be found in Supplementary Tables S2 and S3.

### 3.2. Quality Assessment

All 29 cross-sectional studies had clear data sources and criteria for inclusion or exclusion, and the subjects were from the population. There was no missing data in these studies, and the response rates of the subjects were complete. Two studies [23, 24] explained the reasons for excluding any patient. Three studies [23, 24] reported measures taken to control confounding factors. One study [37] had follow-up but did not report the results. But 3 [16, 27, 30] studies did not report the time that studies started and ended. Neither did these studies report whether the evaluators' subjective factors had any effect on the results, nor did they report measures taken to ensure the quality of the main outcome indicators. The overall quality of the above 29 cross-sectional studies is low, and potential bias may exist. The details of the quality assessment of 8 case-control studies are shown in Table 2.

### 3.3. Meta-Analysis of the Distribution of TCM Constitution in Patients with HUA or Gout

#### 3.3.1. PDC

Twenty-six studies [9–33, 45] containing 27526 patients with HUA reported the distribution of PDC. Because *I*^2^ = 97.6% and *P*=0.000, the random-effects model was used for analysis and the result showed that the proportion of PDC in patients with HUA was 24% (95% CI = (0.20, 0.27), Test of ES = 1: *z* = 13.97, *P*=0.000).

**Figure 2:**
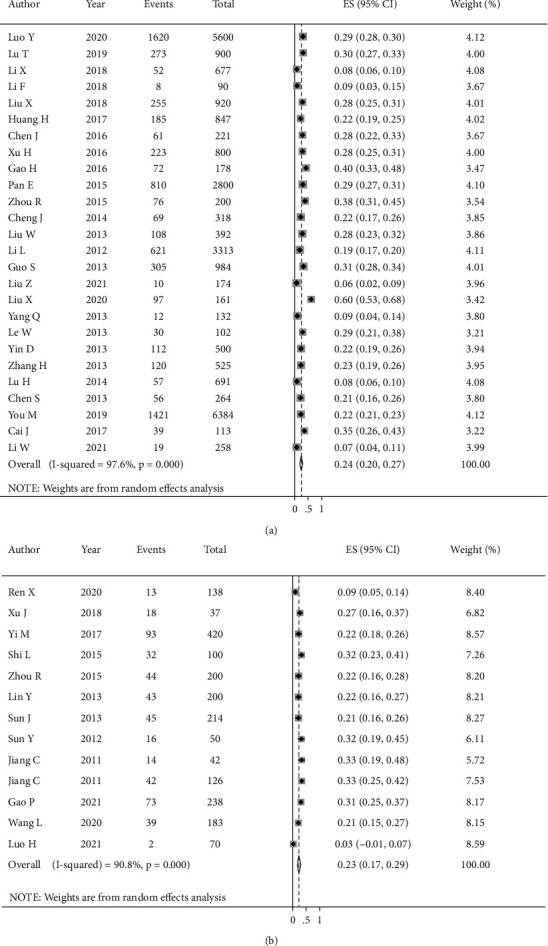
Proportions of PDC in target populations. (a) The proportion of PDC in the population with HUA. (b) The proportion of DHC in the population with gout.

Thirteen studies [18, 34–44, 46] containing 2048 patients with gout reported the distribution of PDC. Because *I*^2^ = 90.8% and *P*=0.000, the random-effects model was used for analysis and the result showed that the proportion of PDC in patients with gout was 23% (95% CI = (0.17, 0.29), Test of ES = 1: *z* = 7.95, *P*=0.000).  Figure 2 represents the proportions of PDC in patients with HUA and gout.

#### 3.3.2. DHC

Twenty-five studies [9–30, 32, 33, 45] containing 27383 patients with HUA reported the distribution of DHC. Because *I*^2^ = 99.3% and *P*=0.000, the random-effects model was used for analysis and the result showed that the proportion of DHC in patients with HUA was 22% (95% CI = (0.16, 0.27), Test of ES = 1: *z* = 7.76, *P*=0.000).

**Figure 3:**
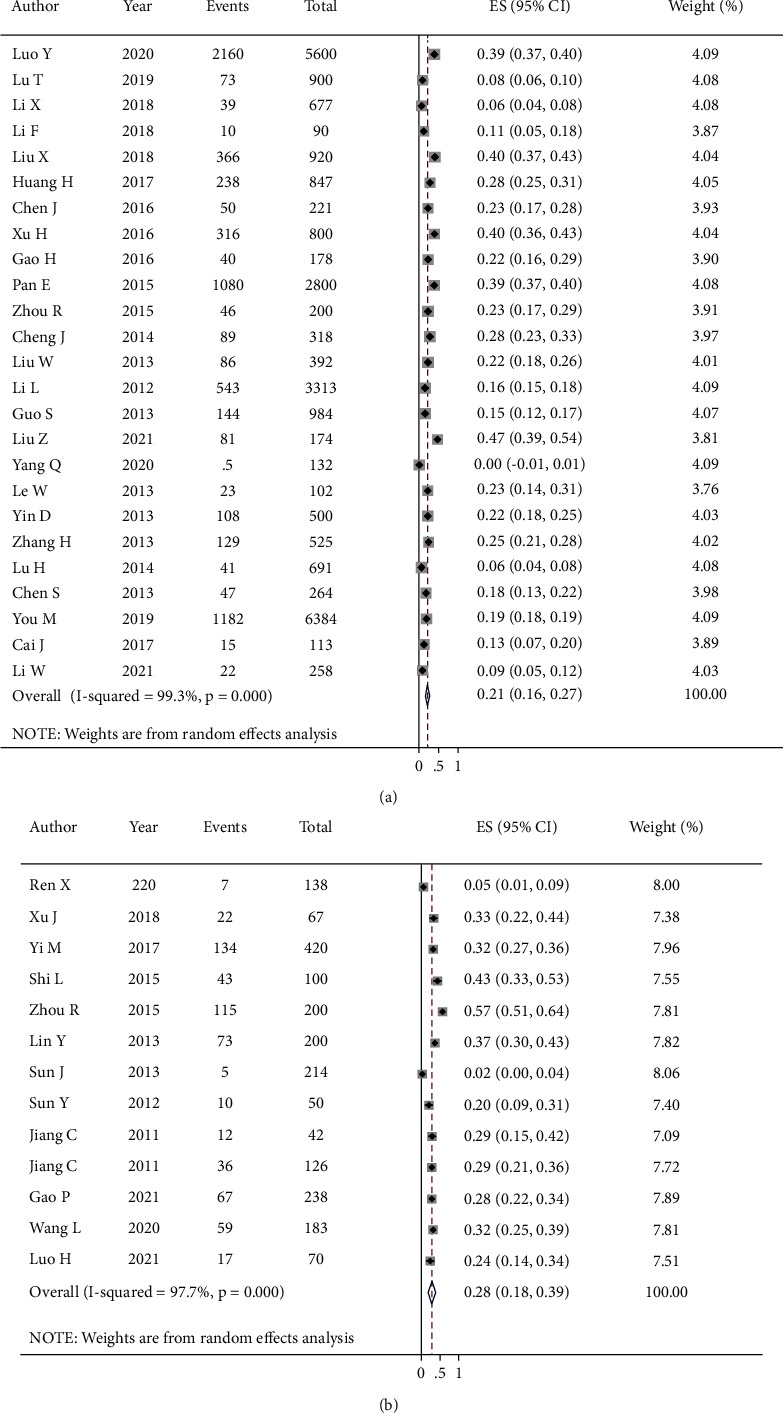
Proportions of DHC in target populations. (a) The proportion of DHC in the population with HUA. (b) The proportion of DHC in the population with gout.

Thirteen studies [18, 34–44, 46] containing 2048 patients with gout reported the distribution of DHC. Because *I*^2^ = 97.7% and *P*=0.000, the random-effects model was used for analysis and the result showed that the proportion of DHC in patients with gout was 28% (95% CI = (0.18, 0.39), Test of ES = 1: *z* = 5.38, *P*=0.000).  Figure 3 represents the proportions of DHC in patients with HUA and gout.

#### 3.3.3. QDC

Twenty-six studies [9–33, 45] containing 27526 patients with HUA reported the distribution of QDC. Because *I*^2^ = 97.9% and *P*=0.000, the random-effects model was used for analysis and the result showed that the proportion of DHC in patients with HUA was 15% (95% CI = (0.12, 0.18), Test of ES = 1: *z* = 11.34, *P*=0.000).

Ten studies [18, 34–36, 38–40, 43, 44, 46] containing 1445 patients with gout reported the distribution of QDC. Because *I*^2^ = 86.4% and *P*=0.000, the random-effects model was used for analysis and the result showed that the proportion of DHC in patients with Gout was 6% (95% CI = (0.03, 0.08), Test of ES = 1: *z* = 4.00, *P*=0.000).

### 3.4. Other Types of TCM Constitution

The proportions of other types of patients with HUA included in this review were BC, YADC, BSC, YIDC, QSC, and ISC, in descending order. And the proportions of other types of patients with gout included in this review were BSC, YADC, BC, BC, YIDC, QSC, and ISC, in descending order. Details are shown in Table 3.

### 3.5. The Distribution of TCM Constitution in Patients with HUA or Gout in Different Regions

Studies were carried out in China. Cases in 1 study about HUA [20] were derived from databases in south China, north China, east China, and southwest China and could not be divided by area. Areas that other studies carried out on HUA included Guangdong, Guangxi, Fujian, Anhui, Zhejiang, Yunnan, Xinjiang's 7 provinces, and Tianjin's 1 municipality directly under the Central Government. Areas that carried out studies on gout included Jilin. Guangdong, Yunnan, Zhejiang, Henan, Shandong's 6 provinces, and Tianjin's 1 municipality are directly under the Central Government. According to the above areas, 6 subgroups that are south China, north China, east China, southwest China, northeast, and northwest China were divided to analyze the proportions of the distribution of TCM constitution in patients with HUA or gout.

In south China, the results of 15 studies [10–13, 15, 17, 19, 20, 22, 24, 25, 27, 28, 30–32] on HUA and 5 studies [37, 38, 41, 42, 44] on gout showed that 3 types of TCM constitution with the highest proportions of patients with HUA were PDC 25% (95% CI = (0.209, 0.291)), DHC 21% (95% CI = (0.130, 0.291)), and QDC 16% (95% CI = (0.118, 0.194)), while the 3 types of TCM constitution with the highest proportions in patients with gout were DHC 34% (95% CI = (0.292, 0.388)), PDC 27% (95% CI = (0.213, 0.328)), and BSC 18% (95% CI = (0.143, 0.223)).

In east China, the results of 5 studies [37, 38, 41, 42, 44] on HUA and 3 studies [40, 43, 46] on gout showed that 3 types of TCM constitution with the highest proportions of patients with HUA were DHC 29% (95% CI = (0.188, 0.393)), PDC 22% (95% CI = (0.131, 0.306)), and QDC 15% (95% CI = (0.093, 0.205)), while 3 types of TCM constitution with the highest proportions of patients with gout were DHC 26% (95% CI = (0.21, 0.31)), PDC 21% (95% CI = (0.00, 0.43)), and BC 14% (95% CI = (0.00, 0.32)).

In southwest China, 2 studies [18, 23] on HUA showed that PDC, DHC, and YADC were the three types of TCM constitution with the highest proportions. In north China and northwest China, 2 studies [16, 26] on HUA showed that PDC, DHC, and YADC were the three types of TCM constitution with the highest proportions. In northeast China, 2 studies [35, 36] on gout showed that DHC, PDC, and YADC were the three types of TCM constitution with the highest proportions.

### 3.6. The Distribution of TCM Constitution in Population with HUA or Gout in Gender

Six studies [14, 16, 19, 23, 26, 30] reported the distribution of TCM constitution in patients with HUA by gender. Meta-analysis showed that the main types of TCM constitution in male patients with HUA were PDC 27% (22%–32%), DHC 26% (19%–32%), and QDC 13% (7%–19%). While in female patients with HUA were YADC 20% (15%–24%), PDC 19% (12%–16%), and QDC 13% (6%–20%). There was no difference in the distribution of PDC (OR = 1.67, 95% CI = (0.98, 2.85)) and QDC (OR = 1.08, 95% CI = (0.64, 1.83)) by gender. But the proportion of men with DHC was 3.1 times more than that of women with DHC (OR = 3.10, 95% CI = (1.61, 5.94)).

Three studies [38, 40, 42] reported the distribution of TCM constitution in gout patients with gender specified. Meta-analysis showed that the main types of TCM constitution in both male and female patients with gout were DHC, PDC, and BSC and showed no difference.

### 3.7. Meta-Analysis of the Comparison of TCM Constitution between HUA or Gout and the Healthy Groups

Ten studies [9, 20–22, 24, 25, 28, 33, 45] reported the distribution of types of TCM constitution in HUA and healthy groups. Meta-analysis showed that PDC and DHC might be risk factors for HUA, while BC and YADC might be protective factors. Four studies [38, 40, 42, 46] reported the distribution of types of TCM constitution in gout and healthy groups. Meta-analysis showed that PDC, DHC, and BSC might be risk factors for gout, while QDC and BC might be protective factors for gout. Details are shown in Table 4.

### 3.8. Assessment of the Stability and Reliability of Meta-Analysis Results

Sensitivity analysis was applied by evaluating the effect value of other studies after eliminating the results of every single study one by one. Sensitivity analysis showed 5 studies [10, 11, 17, 20, 26] had an impact on the stability of the proportion of PDC in patients with HUA and 6 studies [10, 11, 17, 20, 24, 27] had an impact on the stability of the proportion of DHC in patients with HUA. One study [11] influenced the stability of the proportion of QDC in HUA groups. One study [10, 11] had an influence on the proportion of DHC in patients with gout; 1 study [34] had an impact on the proportion of PDC in gout groups; and 1 study [18] influenced the proportion of BSC in gout groups. Details are shown in Supplementary Figure S1.

Sensitivity analysis showed that the main types of TCM constitution in patients with HUA or gout did not change, and the results of the comparison between HUA or gout groups and healthy groups did not change either.

### 3.9. Potential Publication Bias

**Figure 4:**
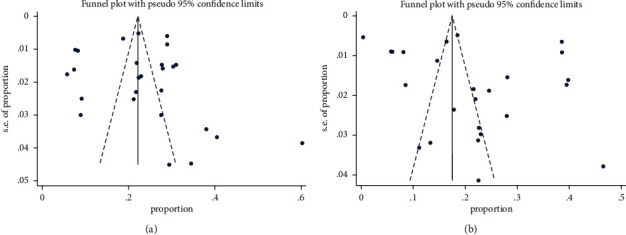
Funnel plots of proportions of the PDC and DHC in target populations. (a) The funnel plot of the proportion of PDC in the population with HUA. (b) The funnel plot of the proportion of DHC in the population with HUA.

The funnel plots and Egger's test were used to detect the publication bias of the proportions of the distribution of each type of TCM constitution in the HUA or gout population. There may be publication bias in proportions of BC, QDC, YADC, and YIDC 4 types of TCM constitution in patients with HUA according to Egger's test (*P* < 0.05). And there may be publication bias in proportions of BC, QDC, QSC, PDC, DHC, ISC, BSC, and YIDC 8 types of TCM constitution in patients with gout according to Egger's test (*P* < 0.05).  Figure 4 contains representative funnel plots that show the proportions of PDC and DHC in the population with HUA are symmetrical. The results of Egger's test are listed in Supplementary Table S4.

## 4. Discussion

### 4.1. Analysis of Types of the TCM Constitution in HUA or Gout Population

The proportions of PDC and DHC in HUA or gout populations in this review were significantly higher than that of the Chinese healthy population, compared to a study in 2009 [47], in which the proportions of PDC and DHC were 7.32% and 9.08%, respectively. The proportion of BSC in the gout population was 8.10%, which was higher than that of the healthy population, while there was no difference in the QDC of the HUA groups compared with the 13.42% of the healthy population. The respective proportions of PDC and DHC in the HUA population were 1.93 times and 2.14 times higher than that of the healthy population, while the proportions of BC and YADC were 0.58 and 0.49 in the healthy population. The proportions of PDC, DHC, and BSC in the gout population were 3.59 times, 4.85 times, and 4.35 times that of the healthy population, while the proportions of BC and QDC were 0.13 and 0.45 of the healthy groups. All the above results were statistically significant, which suggests that PDC, DHC, and QDC are the main types of TCM constitution in the HUA population, and DHC, PDC, and BSC are the main types of TCM constitution in the gout population. Among these, PDC and DHC may be the risk factors for HUA, and PDC, DHC, and BSC may be the risk factors for gout.

The distributions of TCM constitution types are different in different regions [47]. Compared with a large sample cross-sectional study of 108015 samples, YDC (19.3%), QDC (14.1%), and DHC (9.4%) are the three TCM constitutions with the highest proportions in South China, while YDC (23.2%), QDC (12.9%), and DHC (9.2%) are the most three TCM constitutions in East China [48]. In our study, according to the results of subgroup analysis in different regions, both PDC and DHC were the main TCM constitution types in HUA or gout populations in different regions, which confirms that PDC and DHC are closely related to HUA or gout. In terms of the distribution by gender, there was no significant difference in the distribution of DHC, PDC, and BSC in male or female populations with gout. This may be because the pathophysiological mechanism of gout is not associated with gender. As for HUA, PDC, and QDC, there was no difference in the distribution of male and female populations, while men with DHC were much more numerous than women with DHC. This may be because men have an overall higher basal metabolism than women, and drinking alcohol is more common in men than in women [49], while alcohol use is positively correlated with PDC and DHC [50].

### 4.2. The Relationship between PDC, DHC, and HUA

Increased production of uric acid and decreased excretion of uric acid are the main reasons underlying the pathology of HUA. Glutamine is one of the most important precursor substances in the pathway of purine production and determines the rate of uric acid production. A study [51] on the metabolomics of the population with DHC found that the levels of glutamine increased significantly in these people. DHC may promote the synthesis of uric acid through the pathway of purine production. About 2/3 of uric acid is excreted through the kidneys, and the remaining 1/3 is excreted through extrarenal pathways such as the intestine and biliary tract. In recent years, the excretion of uric acid through the intestine has attracted much attention. DHC is related to intestinal microbes and blood or saliva metabolites. A study [52] found that the abundance of probiotics was decreased in the intestinal flora of the population with DHC, while the abundance of conditional pathogenic bacteria was increased. Besides, the basal metabolic rate and daily intake of people with DHC were significantly reduced. The imbalance of intestinal microecology is one of the metabolic characteristics of DHC [53].

PDC has the overall characteristics of metabolic disorders, with pathological characteristics such as abnormal distribution of body fat, lipid metabolism disorders, high levels of insulin, and insulin resistance [54], and is related to a variety of metabolic-related diseases. Patients with HUA have abnormal metabolic pathways such as the metabolism of glucose, lipids, energy, and chronic low-grade inflammation induced by changes in the structure of intestinal flora may cause metabolic disorders [55]. Besides, in the view of gut microbial, patients with PDC showed an increased abundance of Bacteroidetes and the prevotella (a conditioned pathogen) and decreased levels of Firmicutes/Bacteroidetes ratio, as well as the Faecalibacterium (a kind of probiotic) [56]. Furthermore, the changed gut microbes in patients with PDC were mainly associated with carbohydrate and amino acid metabolism, mineral absorption, lipopolysaccharide biosynthesis, primary bile acid biosynthesis, and so on. The altered gut microbial composition and function correspond to the clinical findings of HUA [57]. Therefore, the metabolic characteristics of people with PDC or DHC may be the reasons underlying the pathology of HUA.

More importantly, early intervention based on the TCM constitution shows an effect on HUA and gout. For example, compared with potassium sodium hydrogen citrate (Uralyt-U), a Traditional Chinese Medicine prescription that aimed at regulating phlegm-damp displayed a better curative effect on patients with PDC in urinary acid calculus [58]. After diet guidance, lifestyle adjustment, and other interventions according to the TCM constitution, patients with HUA developed a decreased number of gout attacks compared with those without TCM constitution intervention [59]. These studies confirmed the efficiency of intervention based on the TCM constitution in HUA and gout.

### 4.3. Limitations of the Review

The proportions of the distribution of different TCM constitution types in HUA or gout populations were of high heterogeneity, which was related to regions, research objects, diagnostic criteria, and other confounding factors. The TCM constitution is different depending on the region, gender, and other factors, which is the same as reported in the study by Wang et al. [48]. Sensitivity analysis showed that the results were consistent, while a few studies were not in the overall confidence interval. We carefully analysed these studies but could not find out the reason that might influence the reliability of the data.

Studies on HUA or gout were mainly reported in South China, and there was only one study reported in each of North China, Northeast China, and Northwest China. The findings in these 3 regions needed to be verified by more samples. Only a small number of cross-sectional studies or case-control studies met the cross-sectional study evaluation criteria of the AHRQ or the standards of the NOS scale. The overall quality of the included observational studies is low.

## 5. Conclusion

The meta-analysis of 38028 subjects finds that PDC, DHC, and QDC are the main TCM constitution types in patients with HUA, among which PDC and DHC are the risk factors for HUA, and DHC, PDC, and BSC are the main types of TCM constitution for patients with gout and are the risk factors for gout. While BC and YADC are the protective factors for HUA, BC, and QDC are the protective factors for gout. In the clinical field, early intervention and treatment can be applied to HUA or gout according to the TCM constitution. For scientific research, it is recommended that relevant scientific research be carried out in multiple centers to obtain reliable results. Besides, multicrossed techniques can be used to explore the specific correlation between TCM constitution and HUA or gout. Due to the lack of conclusive evidence on TCM constitution and disease occurrence, more prospective cohort studies related to TCM constitution and HUA or gout can be carried out to verify the causality between TCM constitution and HUA or gout.

## Figures and Tables

**Table 1:** Characteristics of studies on HUA and gout.

Study	Areas	Study type	Period	Sample size (total or case/control)	Age (total or case/control)	Gender ratio(M/F)/(case/control)	Constitution
Liu and Chen [9]	Anhui	CC	2019	175/347	73.8 ± 5.9/73.8 ± 5.7	99 : 75/160 : 188	9
Luo et al. [10]	Guangdong	CS	2017-2018	5600	43.7 ± 5.7	3340 : 2260	9
Liuxiao [31]	Guangdong	CC	2016-2017	135/77	41.0/40.0	NR	9
Lu [27]	Guangxi	CS	2017–2019	900	55.3 ± 12.5	623 : 227	9
Li and Chen [11]	Guangdong	CS	2013–2016	677	Men: 40.8 ± 9.7 Women: 46.4 ± 11.5	513 : 164	9
Liufei [12]	Guangdong	CS	2017-2018	90	44.0	52 : 38	9
Liu et al. [29]	Anhui	CS	2016-2017	920	54.3 ± 10.9	722 : 198	9
Huang et al. [14]	Zhejiang	CS	2014–2016	766	59.0 ± 16.0	591 : 175	9
Chen et al. [13]	Guangdong	CS	2014–2016	221	22–55	126 : 95	9
Xu et al. [15]	Guangdong	CS	2014-2015	800	40.3 ± 4.1	620 : 180	9
Gao [16]	Tianjin	CS	2014-2015	210	60–80	150 : 60	5
Pan and Ouyang [17]	Guangdong	CS	2014	2700	42.1 ± 8.6	1620 : 1080	9
Zhourui [18]	Yunnan	CS	2014	200	58.1	136 : 64	9
Cheng and Wu [19]	Guangdong	CS	2012-2013	286	43.3 ± 13.2	208 : 82	9
Lu et al. [20]	Guangdong	CS	2008-2009	6853	33.6 ± 9.7	3630 : 3223	9
Chen Li et al. [21]	Fujian	CS	2009–2011	884	50.8 ± 12.2/50.1 ± 14.7	NR	9
Liuwen [23]	Yunnan	CS	2012	392	59.4 ± 16.1	291 : 101	9
Yangqian [24]	Guangdong	CS	2012	276	NR	NR	9
Lewen [25]	Guangdong	CC	2012-2013	107/102	34.6 ± 9.9/33.9 ± 8.7	68 : 34/39 : 68	9
Li et al. [26]	Xinjiang	CS	2010–2012	1400	55.3 ± 12.5	986 : 414	9
Yin et al. [28]	Guangdong	CC	2011-2012	500/500	42.5 ± 2.4/43.2 ± 2.5	279 : 221	9
Guo et al. [30]	Guangdong	CS	NR	984	39.0 ± 12.3	697 : 287	Balanced, phlegm-dampness, damp-heat, qi-deficiency
Zhang et al. [22]	Guangdong	CC	2010–2012	525/518	55.7 ± 5.1/54.3 ± 6.1	344 : 181/335 : 183	9
You et al. [32]	Guangdong	CS	2011–2016	6384	48.2 ± 16.6	4082 : 1940	9
Cai et al. [33]	Jiangxi	CS	2016-2017	1037	44.7 ± 17.6	616 : 421	9
Liwen [45]	Heilongjiang	CC	2018–2020	258/294	35.0–44.0/34.0–39.0	193 : 65/26 : 268	9
Renxiao [34]	Tianjin	CS	2018–2020	124	39.59	124 : 0	9
Xu et al. [35]	Jilin	CS	2016	67	40.9 ± 13.0	63 : 4	8 (without inherited special)
Yiming [36]	Jilin	CS	2016	420	38.5 ± 10.0	407 : 13	9
Shi et al. [37]	Guangdong	CS	2012-2013	100	50.58	69 : 31	Phlegm-dampness, damp-heat, blood stasis
Zhourui [18]	Yunnan	CS	2014	200	54	179 : 21	9
Lin et al. [38]	Guangdong	CC	NR	200/200	20–65	163 : 37/172 : 28	9
Sun et al. [39]	Zhejiang and Henan	CS	2010–2012	214	40.08	186 : 28	9
Sun et al. [40]	Zhejiang	CC	2010-2011	50/50	51.5	Men only	9
Jiang and Li [41]	Guangdong	CS	2010	42	41.2 ± 8.9	Men only	Phlegm-dampness, damp-heat, blood stasis
Jiangchun [42]	Guangdong	CC	2009-2010	126/42	50.1 ± 8.1/51.4 ± 11.6	40 : 2/119 : 7	Phlegm-dampness, damp-heat, blood stasis
Gao and Feng [43]	Shandong	CS	2013–2019	238	NR	NR	9
Wanglin [44]	Guangdong	CS	2019	183	53.8 ± 16.1	166 : 17	6
Luo et al. [46]	Zhejiang	CC	2020	70/50	40.4 ± 14.9/36.1 ± 11.6	66 : 4/47 : 3	8 (without inherited special)

*Note*. CC: cross-sectional study; CS: case-control study; NR: no record.

**Table 2:** Quality assessment of case-control studies.

Study	Case definition	Representativeness of the cases	Definition of controls	Selection of controls	Control the most important factors	Control any other factor	Ascertainment of exposure	Same method in exposure of cases and controls	Nonresponse rate	Total
Liu and Chen [9]	☆	☆	☆		☆			☆	☆	6
Liuxiao [31]	☆	☆	☆		☆			☆	☆	6
Lewen [25]	☆	☆	☆		☆	☆		☆	☆	7
Yin et al. [28]	☆	☆	☆		☆			☆	☆	6
Zhang et al. [22]	☆	☆	☆		☆			☆	☆	6
Lin et al. [38]	☆	☆	☆		☆			☆	☆	6
Sun et al. [40]	☆	☆	☆					☆	☆	5
Jiangchun [42]	☆	☆	☆		☆			☆	☆	6
Liwen [45]	☆	☆	☆					☆	☆	5
Luo et al. [46]	☆	☆	☆		☆			☆	☆	6

**Table 3:** Meta-analysis of proportions of other types of TCM constitution.

Types of TCM constitution	Disease	Numbers of patients	Proportion (%)	95% CI	Heterogeneity test		*Z*	*P* value
					*I* ^2^ (%)	*P* value		
BC	HUA	27366	12	0.10–0.14	98.3	0.00	12.20	≤0.01
	Gout	1445	9	0.06–0.12	97.6	0.00	6.06	≤0.01

YADC	HUA	26399	9	0.07–0.10	97.0	0.00	9.52	≤0.01
	Gout	1445	10	0.07–0.14	87.6	0.00	5.33	≤0.01

BSC	HUA	26399	7	0.05–0.08	97.8	0.00	7.09	≤0.01
	Gout	2048	11	0.08–0.15	89.1	0.00	6.22	≤0.01

YIDC	HUA	26399	5	0.04–0.06	87.4	0.00	11.18	≤0.01
	Gout	1445	5	0.02–0.07	88.9	0.00	5.33	≤0.01

QSC	HUA	26221	5	0.04–0.06	92.8	0.00	8.79	≤0.01
	Gout	1445	2	0.01–0.03	81.7	0.00	3.62	≤0.01

ISC	HUA	26221	2	0.01–0.04	97.5	0.00	3.98	≤0.01
	Gout	1375	1	0.00–0.02	79.9	0.00	2.23	0.03

**Table 4:** Meta-analysis of the comparison of TCM constitution between HUA or gout and the healthy groups.

TCM constitution	Disease	Patients/health population		OR	95% CI	Heterogeneity test		*Z*	*P* value
		Case groups	General groups			*I* ^2^ (%)	*P* value		
PDC	HUA	552/2920	999/9966	1.93	1.27–2.93	85.8	0.00	3.08	≤0.01
	Gout	103/446	25/342	3.59	1.65–7.80	44.1	0.15	5.32	≤0.01

DHC	HUA	466/2759	607/9617	2.14	1.47–3.13	81.1	0.00	3.94	≤0.01
	Gout	136/446	52/342	4.85	1.62–14.57	66.1	0.03	2.81	≤0.01

QDC	HUA	410/2920	1444/9696	0.83	0.56–1.23	86.8	0.00	0.91	0.36
	Gout	13/320	27/300	0.45	0.23–0.89	0.0	0.94	2.31	0.02

BC	HUA	645/2920	3331/9695	0.58	0.37–0.90	92.0	0.00	2.40	0.02
	Gout	36/320	113/300	0.13	0.02–0.74	90.6	0.00	2.31	0.02

YADC	HUA	249/2759	1488/9617	0.49	0.36–0.67	68.3	0.00	4.41	≤0.01
	Gout	17/320	28/300	0.54	0.24–1.23	16.7	0.30	1.88	0.06

BSC	HUA	223/2770	398/9618	1.18	0.73–1.90	72.9	0.00	0.68	0.50
	Gout	69/446	13/342	4.35	2.33–8.11	0.0	0.96	4.66	≤0.01

YIDC	HUA	157/2759	750/9620	0.74	0.54–1.01	44.7	0.07	2.79	≤0.01
	Gout	17/320	26/300	0.55	0.29–1.08	0.0	0.43	1.85	0.06

QSC	HUA	124/2759	627/9620	0.75	0.54–1.04	34.9	0.14	2.84	≤0.01
	Gout	24/320	14/300	1.55	0.40–5.99	40.1	0.19	1.55	0.12

ISC	HUA	63/2759	295/9617	1.00	0.71–1.41	4.5	0.40	0.23	0.82
	Gout	1/250	4/250	0.54	0.02–14.50	56.3	0.13	0.37	0.71

## Data Availability

The datasets generated during and/or analysed during the current study are available from the corresponding author upon reasonable request.

## Supplementary Materials

Supplementary Materials Table. S1 Search strategies of the English databases. Table. S2 The number of people in each type of the TCM constitution according to each study. Table S3 Comparative data of each type of the TCM constitution. Table. S4 The results of Egger's test. Figure. S1 Sensitivity analysis of the distribution of the main types of the TCM constitution.
