# Supplementary material for: The Correlation between Traditional Chinese Medicine Constitution and Hyperuricemia and Gout: A Systematic Review and Meta-Analysis
Source: Evid Based Complement Alternat Med. 2023 Apr 17;2023:5097490. doi: 10.1155/2023/5097490 (PMC10125745; doi:10.1155/2023/5097490)
Supplement: Supplementary Materials — Table. S1 Search strategies of the English databases. Table. S2 The number of people in each type of the TCM constitution according to each study. Table S3 Comparative data of each type of the TCM constitution. Table. S4 The results of Egger's test. Figure. S1 Sensitivity analysis of the distribution of the main types of the TCM constitution. [file 5097490.f1.docx]

**Supplementary material**

1. **Table S1** Search strategies of the English databases

| Database | Search strategy |
| --- | --- |
| Pubmed | ((hyperuricemia) OR (gout) OR (gouts)) AND (constitution) AND (chinese medicine) |
| Web of Science | (TS=(hyperuricemia) OR TS=(gout) OR TS=(gouts)) AND TS=(constitution) AND TS=(chinese medicine) |
| Cochrane Library | hyperuricemia or gout in Title Abstract Keyword AND constitution in Title Abstract Keyword AND chinese medicine in All Text |
| Embase | ((hyperuricemia) OR (gout) OR (gouts)) AND (constitution) AND (chinese medicine) |

1. **Table S2** The number of people in each type of the TCM constitution according to each study

| Disease | Research | The number of people in each type of TCM constitution | | | | | | | | |
| --- | --- | --- | --- | --- | --- | --- | --- | --- | --- | --- |
|  |  | YIDC | PDC | DHC | BSC | BC | QDC | YADC | QSC | ISC |
| HUA | Luo Y 2020 | 368 | 1620 | 2160 | 278 | 80 | 540 | 180 | 202 | 172 |
|  | Lu T 2019 | 25 | 273 | 73 | 38 | 35 | 188 | 149 | 107 | 12 |
|  | Li X 2018 | 56 | 52 | 39 | 5 | 115 | 364 | 26 | 10 | 10 |
|  | Li F 2018 | 1 | 8 | 10 | 7 | 52 | 5 | 5 | 2 | 0 |
|  | Liu X 2018 | 61 | 255 | 366 | 44 | 25 | 80 | 30 | 33 | 26 |
|  | Huang H 2017 | 27 | 185 | 238 | 70 | 45 | 107 | 93 | 74 | 8 |
|  | Chen J 2016 | 8 | 61 | 50 | 24 | 15 | 31 | 14 | 17 | 1 |
|  | Xu H 2016 | 53 | 223 | 316 | 39 | 22 | 70 | 26 | 28 | 23 |
|  | Gao H 2016 | - | 72 | 40 | 11 | - | 20 | 35 | - | - |
|  | Pan E 2015 | 184 | 810 | 1080 | 139 | 40 | 270 | 90 | 101 | 86 |
|  | Zhou R 2015 | 6 | 76 | 46 | 8 | 10 | 20 | 30 | 4 | 0 |
|  | Cheng J 2014 | 10 | 69 | 89 | 30 | 17 | 40 | 35 | 26 | 2 |
|  | Liu W 2013 | 21 | 108 | 86 | 25 | 31 | 35 | 60 | 17 | 9 |
|  | Li L 2012 | 236 | 621 | 543 | 413 | 160 | 358 | 394 | 231 | 357 |
|  | Guo S 2013 | - | 305 | 144 | - | 185 | 281 | - | - | - |
|  | Liu Z 2021 | 14 | 10 | 81 | 46 | 7 | 9 | 4 | 2 | 1 |
|  | Liu X 2020 | - | 97 | - | - | 4 | 29 | - | - | - |
|  | Yang Q 2013 | 4 | 12 | 0 | 0 | 68 | 23 | 18 | 5 | 2 |
|  | Le W 2013 | 1 | 30 | 23 | 0 | 14 | 17 | 16 | 1 | 0 |
|  | Yin D 2013 | 27 | 112 | 108 | 83 | 56 | 45 | 34 | 22 | 13 |
|  | Zhang H 2013 | 38 | 120 | 129 | 58 | 45 | 33 | 39 | 44 | 19 |
|  | Lu H 2014 | 52 | 57 | 41 | 18 | 285 | 94 | 87 | 33 | 24 |
|  | Chen S 2013 | 10 | 56 | 47 | 13 | 53 | 59 | 17 | 8 | 0 |
|  | You M 2019 | 452 | 1421 | 1182 | 834 | 362 | 328 | 687 | 465 | 653 |
|  | Cai J 2017 | 2 | 39 | 15 | 2 | 15 | 36 | 1 | 2 | 1 |
|  | Li W 2021 | 9 | 19 | 22 | 2 | 98 | 65 | 33 | 7 | 3 |
| Gout | Ren X 2020 | 3 | 13 | 7 | 4 | 93 | 6 | 8 | 3 | 1 |
|  | Xu J 2018 | 3 | 18 | 22 | 2 | 4 | 6 | 10 | 2 | 0 |
|  | Yi M 2017 | 22 | 93 | 134 | 25 | 30 | 35 | 61 | 17 | 3 |
|  | Shi L 2015 | **-** | 32 | 43 | 25 | **-** | **-** | **-** | **-** | **-** |
|  | Zhou R 2015 | 10 | 44 | 115 | 5 | 10 | 2 | 11 | 1 | 2 |
|  | Lin Y 2013 | 6 | 43 | 73 | 29 | 6 | 10 | 13 | 20 | 0 |
|  | Sun J 2013 | 38 | 45 | 5 | 23 | 0 | 33 | 27 | 10 | 33 |
|  | Sun Y 2012 | 2 | 16 | 10 | 9 | 4 | 2 | 2 | 4 | 1 |
|  | Jiang C 2011 | **-** | 14 | 12 | 9 | **-** | **-** | **-** | **-** | **-** |
|  | Jiang C 2011 | **-** | 42 | 36 | 27 | **-** | **-** | **-** | **-** | **-** |
|  | Gao P 2021 | 0 | 73 | 67 | 40 | 0 | 23 | 35 | 0 | 0 |
|  | Wang L 2020 | 4 | 39 | 59 | 28 | 0 | 4 | 49 | 0 | 0 |
|  | Luo H 2021 | 5 | 2 | 17 | 4 | 26 | 1 | 2 | 0 | **-** |

1. **Table S3** Comparative data of each type of the TCM constitution

| Study | Comparative groups | The number of people in each type of TCM constitution | | | | | | | | |
| --- | --- | --- | --- | --- | --- | --- | --- | --- | --- | --- |
|  |  | YIDC | PDC | DHC | BSC | BC | QDC | YADC | QSC | ISC |
| Liu Z  2021 | HUA | 14 | 10 | 81 | 46 | 7 | 9 | 4 | 2 | 1 |
|  | Health | 65 | 36 | 39 | 54 | 28 | 43 | 60 | 20 | 3 |
| Yang Q  2013 | HUA | 4 | 12 | 0 | 0 | 68 | 23 | 18 | 5 | 2 |
|  | Health | 5 | 10 | 1 | 0 | 74 | 25 | 24 | 4 | 1 |
| Le W  2013 | HUA | 1 | 30 | 23 | 0 | 14 | 17 | 16 | 1 | 0 |
|  | Health | 0 | 8 | 13 | 0 | 14 | 44 | 24 | 1 | 3 |
| Yin D  2013 | HUA | 27 | 112 | 108 | 83 | 56 | 45 | 34 | 22 | 13 |
|  | Health | 43 | 56 | 50 | 42 | 143 | 62 | 76 | 18 | 10 |
| Zhang H  2013 | HUA | 38 | 120 | 129 | 58 | 45 | 33 | 39 | 44 | 19 |
|  | Health | 39 | 88 | 78 | 30 | 109 | 72 | 48 | 40 | 14 |
| Lu H  2014 | HUA | 52 | 57 | 41 | 18 | 285 | 94 | 87 | 33 | 24 |
|  | Health | 487 | 254 | 269 | 197 | 2576 | 610 | 1040 | 472 | 257 |
| Chen S  2013 | HUA | 10 | 56 | 47 | 13 | 53 | 59 | 17 | 8 | 0 |
|  | Health | 32 | 120 | 42 | 50 | 131 | **0** | 88 | 32 | 2 |
| Cai J  2017 | HUA | 2 | 39 | 15 | 2 | 15 | 36 | 1 | 2 | 1 |
|  | Health | 72 | 150 | 96 | 11 | 116 | 412 | 43 | 24 | 0 |
| Li W  2021 | HUA | 9 | 19 | 22 | 2 | 98 | 65 | 33 | 7 | 3 |
|  | Health | 7 | 6 | 19 | 13 | 94 | 49 | 85 | 16 | 5 |
| Lin Y  2013 | Gout | 6 | 43 | 73 | 29 | 6 | 10 | 13 | 20 | 0 |
|  | Health | 16 | 17 | 47 | 8 | 54 | 22 | 21 | 11 | 4 |
| Sun Y  2012 | Gout | 2 | 16 | 10 | 9 | 4 | 2 | 2 | 4 | 1 |
|  | Health | 3 | 2 | 2 | 2 | 35 | 4 | 1 | 1 | 0 |
| Jiang C  2011 | Gout | Com | 42 | 36 | 27 | Com | Com | Com | Com | Com |
|  | Health | 1 | 4 | 2 | 2 | 28 | 2 | 2 | 1 | - |
| Luo H  2021 | Gout | 5 | 2 | 17 | 4 | 26 | 1 | 2 | 0 | 0 |
|  | Health | 6 | 2 | 1 | 1 | 24 | 1 | 6 | 2 | 0 |

Note: Com: The number of YIDC, BC, QDC, YADC, QSC, and ISC was combined in total of 21 in the study, without exact numbers of each TCM constitution.

1. **Table S4** Results of Egger’s test

| The type of TCM constitution | Disease | Slope bias |
| --- | --- | --- |
|  |  | P>\|t\| |
| YIDC | HUA | 0.022 |
|  | Gout | 0.003 |
| PDC | HUA | 0.774 |
|  | Gout | 0.023 |
| DHC | HUA | 0.299 |
|  | Gout | 0.002 |
| BSC | HUA | 0.270 |
|  | Gout | 0.001 |
| BC | HUA | 0.000 |
|  | Gout | 0.012 |
| QDC | HUA | 0.002 |
|  | Gout | 0.018 |
| YADC | HUA | 0.023 |
|  | Gout | 0.197 |
| QSC | HUA | 0.918 |
|  | Gout | 0.002 |
| ISC | HUA | 0.316 |
|  | Gout | 0.029 |

1. **Figure. S1**

**b**

**a**

**c**

**d**

**f**

**e**

**Figure. S1** Sensitivity analysis of the distribution of the main types of TCM constitution. **a** Sensitivity analysis of the distribution of PDC in population with HUA. **b** Sensitivity analysis of the distribution of DHC in population with HUA. **c** Sensitivity analysis of the distribution of QDC in population with HUA. **d** Sensitivity analysis of the distribution of PDC in population with gout. **e** Sensitivity analysis of the distribution of DHC in population with gout. **f** Sensitivity analysis of the distribution of BSC in population with gout.
